# A novel de novo truncating TRIM8 variant associated with childhood-onset focal segmental glomerulosclerosis without epileptic encephalopathy: a case report

**DOI:** 10.1186/s12882-021-02626-1

**Published:** 2021-12-20

**Authors:** Yoko Shirai, Kenichiro Miura, Naoto Kaneko, Kiyonobu Ishizuka, Amane Endo, Taeko Hashimoto, Shoichiro Kanda, Yutaka Harita, Motoshi Hattori

**Affiliations:** 1 Department of Pediatric Nephrology, Tokyo Women’s Medical University, Tokyo, Japan; 2 Department of Pediatrics and Adolescent Medicine, Juntendo University Graduate School of Medicine, Tokyo, Japan; 3 Department of Pediatrics, Yamagata University School of Medicine, Yamagata, Japan; 4 Department of Pediatrics, Graduate School of Medicine, The University of Tokyo, Tokyo, Japan

**Keywords:** Focal segmental glomerulosclerosis, Nonsense-mediated mRNA decay, Suppressor of cytokine signaling 1, Tripartite motif containing 8, Case report

## Abstract

**Background:**

Heterozygous truncating variants in the *Tripartite motif containing 8* (*TRIM8*) gene have been reported to cause epileptic encephalopathy, both with and without proteinuria. A recent study showed a lack of TRIM8 protein expression, with suppressor of cytokine signaling 1 (SOCS1) overexpression, in podocytes and tubules from a patient with a *TRIM8* variant, who presented with epileptic encephalopathy and focal segmental glomerulosclerosis (FSGS). To date, no patients with *TRIM8* variants who presented with nephrotic syndrome but without neurological manifestations have been described.

**Case presentation:**

An 8-year-old girl presented with nephrotic syndrome, without epilepsy or developmental delay. Her kidney biopsy specimens showed FSGS and cystic dilatations of the distal tubules. Whole-exome sequencing identified a novel de novo heterozygous variant in the C-terminal encoding portion of *TRIM8* (c.1461C > A), resulting in a premature stop codon (p.Tyr487*). Reverse transcription-polymerase chain reaction using peripheral blood mononuclear cells identified the mRNA sequence of the mutant allele, which confirmed an escape from nonsense-mediated mRNA decay. Immunofluorescence studies showed a lack of TRIM8 expression in glomerular and tubular cells and cystic dilatation of distal tubules. Immunohistochemical studies showed overexpression of SOCS1 in glomerular and tubular cells.

**Conclusions:**

We reported a patient with FSGS, associated with a de novo heterozygous *TRIM8* variant, without any neurological manifestations. Our results expanded the clinical phenotypic spectrum of *TRIM8* variants.

**Supplementary Information:**

The online version contains supplementary material available at 10.1186/s12882-021-02626-1.

## Background

Focal segmental glomerulosclerosis (FSGS) describes a histologic lesion that causes podocyte injury. To date, More than 50 causative genes have been associated with genetic forms of FSGS [1, 2]

Sakai et al. described a 10-year-old boy with epileptic encephalopathy, severe developmental delay, and a de novo variant the *tripartite motif containing 8* (*TRIM8*) gene [3]. Assoum et al. described five additional individuals of childhood-onset epileptic encephalopathy associated with heterozygous de novo variants in the C-terminus-encoding portion of *TRIM8*, including three individuals who presented with proteinuria [4]. Recently, Warren et al. reported an individual with epileptic encephalopathy and nephrotic syndrome with a *TRIM8* variant, and his kidney specimen showed FSGS [5]. They also performed immunohistochemical (IHC) staining, using an anti-TRIM8 antibody, and demonstrated a lack of TRIM8 protein expression, with suppressor of cytokine signaling 1 (SOCS1) overexpression, which is regulated by TRIM8, in the podocytes and tubules [5]. In addition, McClatchey et al. presented an individual with FSGS which required renal replacement therapies, but only mild neurodevelopmental problems [6].

Here, we report a patient with a novel, de novo, heterozygous nonsense variant in the last exon of *TRIM8,* who presented with nephrotic range proteinuria and progressed to end-stage renal disease (ESRD) but did not present with any neurological manifestations. A kidney biopsy showed FSGS and cystic dilatations of the tubules. To evaluate pathogenicity of the patient’s nonsense *TRIM8* variant and characterize the pathological changes in glomerular and tubulointerstitial lesions, we performed mRNA analysis in mononuclear cells and immunofluorescence studies on kidney specimens from the present patient and control samples. In addition, the loci of *TRIM8* nonsense variants and clinical manifestations in previously reported individuals and the present patient were reviewed.

## Case presentation

**Fig. 1:**
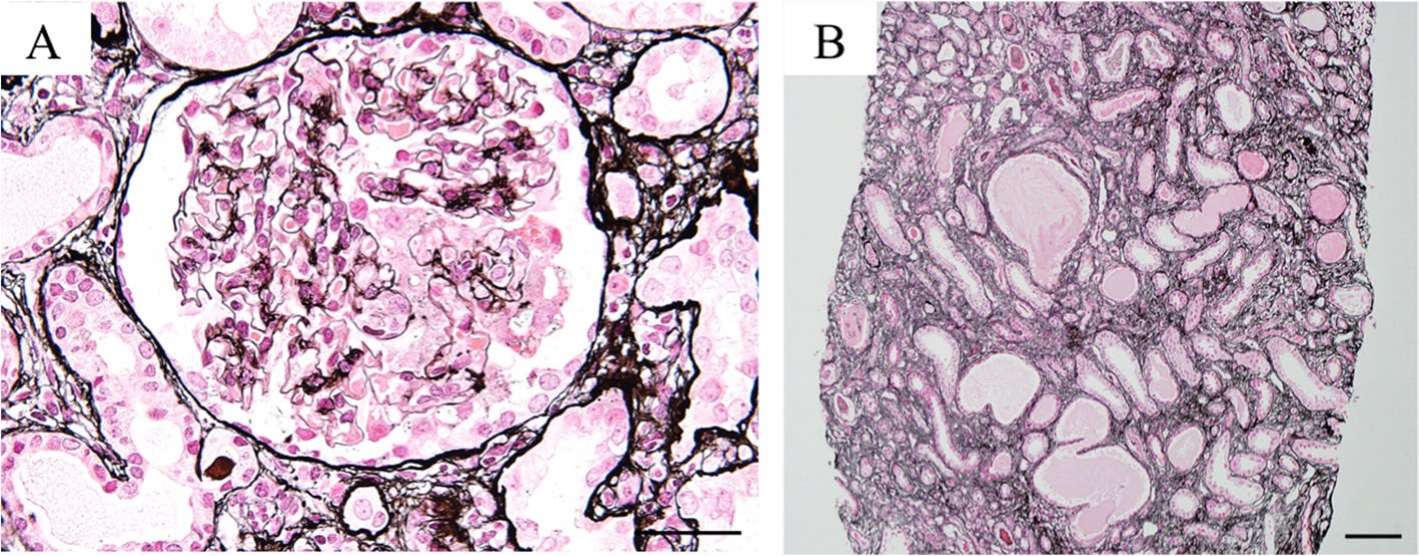
Pathological findings of the kidney specimen obtained from the present patient. (**A**) The glomeruli in kidney specimens obtained from the present patient showed cellular lesions, characterized by swollen, vacuolated, and proliferative glomerular epithelial cells throughout Bowman’s space. The underlying glomerular capillaries were partially collapsed and occluded by swollen endothelial cells and karyorrhexis (original magnification × 400. Scale bar = 50 µm); (**B**) Cystic dilatations of the tubules were also observed (original magnification × 200. Scale bar = 50 µm)

A girl presented with asymptomatic proteinuria, which was revealed by a urinary screening test performed in Japan when she was 3 years old. She had no family history of renal or neurological disorders. The urine protein to creatinine ratio (UPCR) was 1.0–1.5 g/gCr (reference range < 0.2 g/gCr) at that time. Ultrasonography revealed normal echogenicity in both kidneys. She developed nephrotic syndrome, without systemic edema, at the age of 8 years. Polyuria and polydipsia with a urine output of 3L in a day were also documented. UPCR was 11.5 g/gCr, and the serum albumin level was 2.1 g/dL (reference range 3.7–5.5 g/dL). The serum creatinine level was 0.97 mg/dL (eGFR was 46.8 mL/min/1.73m^2^). The urine specific gravity was 1.008, and urinary beta 2-microglobulin increased to 9,269 µg/L (reference range ≤ 150 μg/L). A kidney biopsy revealed that 13 (52%) of 25 glomeruli showed segmental or global sclerosis. Furthermore, two glomeruli showed cellular lesions, which were characterized by swollen, vacuolated, and proliferative glomerular epithelial cells, throughout Bowman’s space. The underlying glomerular capillaries were partially collapsed and occluded by swollen endothelial cells and karyorrhexis, which was consistent with a pathological diagnosis of FSGS (Fig. 1A) [7]. No glomeruli with collapse and overlying podocyte hypertrophy and hyperplasia were not observed. Cystic dilatations of the tubules and interstitial fibrosis were also observed (Fig. 1B). The patient presented with no neurological manifestations, such as seizures or developmental delays. Brain magnetic resonance imaging (MRI) and electroencephalogram detected no abnormalities. She could hold her head up at 4 months old, sit at 8 months old, pull up to stand at 9 moths and speak single words at 1 year and 6 months old. She did not need special support to attend school. Her renal function continued to deteriorate, and she eventually developed ESRD, despite the administration of angiotensin receptor blockers. At 9 years of age, pre-emptive kidney transplantation was performed, with a kidney donated by her mother. No recurrence of proteinuria has been observed for 1 year and 9 months after transplantation.

We performed whole-exome analysis using a previously described method [8], focusing on variants in the genes that are currently known to cause FSGS or nephronophthisis (Tables S1 and S2), and identified a de novo novel heterozygous C to A transition (c.1461C > A) in the last exon of *TRIM8*, resulting in a premature stop codon (p.Tyr487*). The alternative and reference allele counts were 68 (46%) and 80 (54%), respectively. Sanger sequencing showed that the individual had the variant but that her parents did not. This variant was absent in population databases including the Exome Aggregation Consortium database (ExAC, http://exac.broadinstitute.org/), Genome Aggregation Database (gnomAD, http://gnomad.broadinstitute.org), 1000 Genomes (1000G, http://asia.ensembl.org/Homo_sapiens/Info/Index), ESP6500 (http://evs.gs.washington.edu/EVS/). No additional pathogenic variants in the genes that are currently known to cause FSGS or nephronophthisis were identified (Tables S1 and S2). This variant was classified as pathogenic (PVS1, PM1, PM2, PM6, and PP4) based on the criteria developed by the American College of Medical Genetics and Genomics [9].

**Fig. 2:**
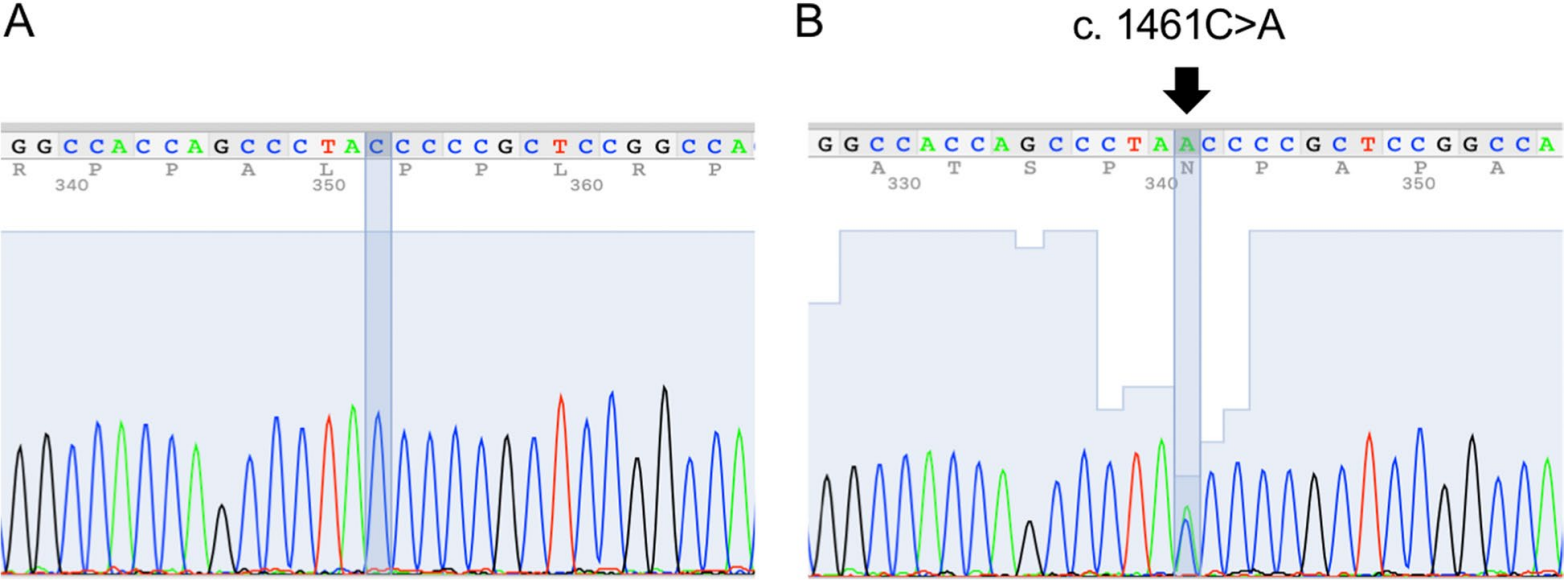
Sequence analysis of mRNA showed mutant allele of the TRIM8 gene. The TRIM8 exon 6 sequences from a control sample (**A**) and the present patient (**B**) are shown. The arrow in panel (**B**) indicates the base substitution, in a heterozygous state. The polymerase chain reaction product was not amplified when RNA samples were treated without reverse transcriptase, which confirmed the lack of genomic DNA contamination

The sequence analysis of mRNA was performed as previouly reported methods [10]. RNA was extracted from peripheral blood mononuclear cells with the RNeasy Mini Kit (QIAGEN), according to the manufacturer’s instructions. The RNA was treated with DNase (QIAGEN) to avoid genomic DNA contamination, and 200 ng of total RNA was reverse transcribed, using the SuperScript VILO cDNA Synthesis Kit (Thermo Fisher Scientific) for the mRNA analysis. The following primers were used to amplify and sequence exon 6 of *TRIM8* from cDNA: 5’-GAGTGTCCCCCTGTACCCTT -3’ (forward) and 5’-CTACAGGGTGTATGGGCAGC-3’ (reverse). Polymerase chain reaction experiments were performed, using Invitrogen Platinum II Taq Hot-Start DNA Polymerase (Thermo Fisher Scientific) and T100TM Thermal Cycler (Bio-Rad Laboratories) identifing mRNA sequences transcribed from the *TRIM8* mutant allele (Fig. 2), which confirmed the escape from nonsense-mediated mRNA decay (NMD) [11].

**Fig. 3:**
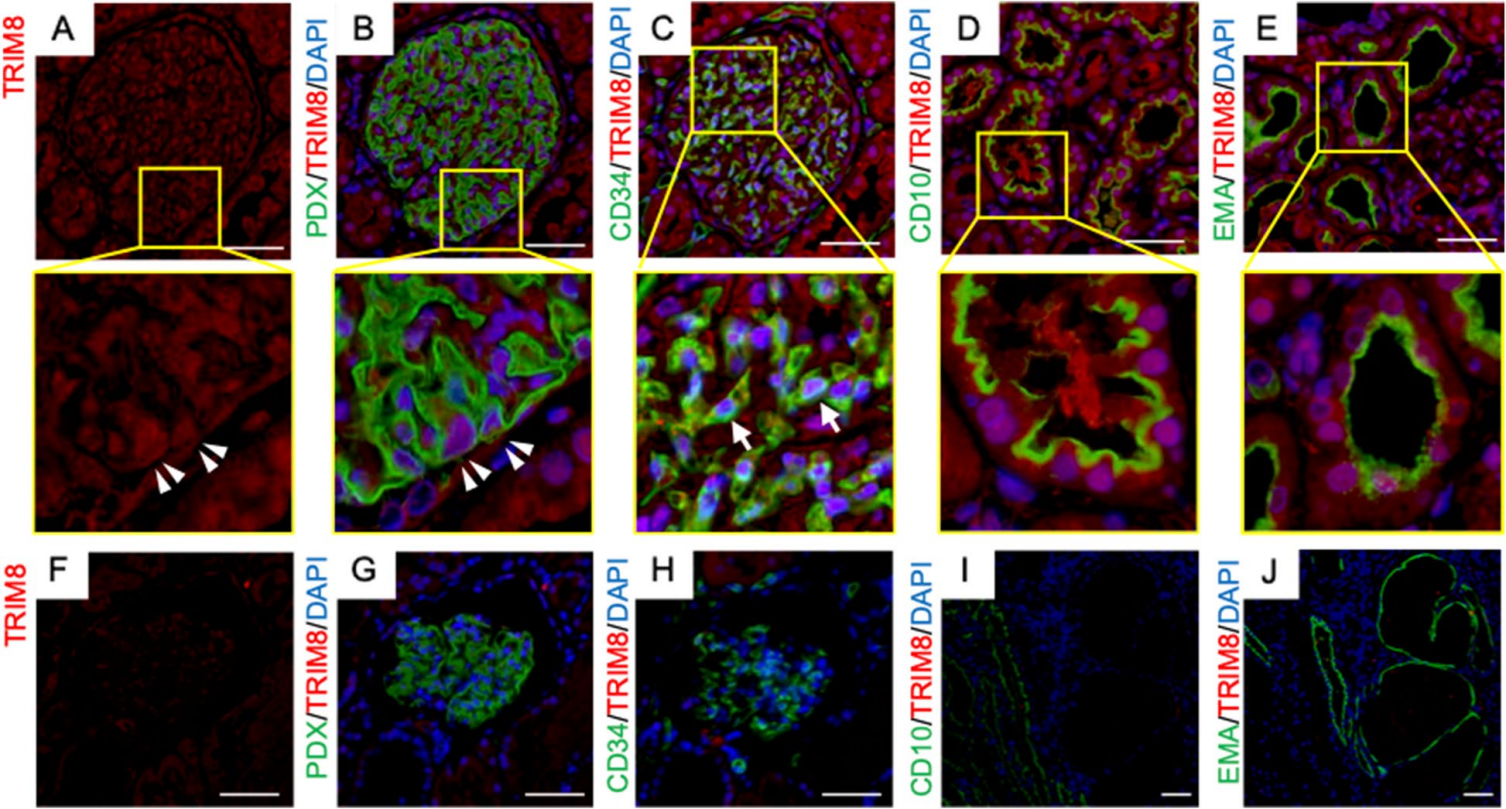
Immunofluorescence staining using anti-TRIM8 antibody. Immunofluorescence staining of the kidney specimen, using an anti-TRIM8 antibody; (**A**) normal control, (**F**) the present patient, combined with anti-podocalyxin antibody, (**B**) normal control, (**G**) the present patient; anti-CD34 antibody, (**C**) normal control, (**H**) the present case; anti-CD10 antibody (**D**) normal control, (**I**) the present patient; and anti-EMA antibody (**E**) normal control, (**J**) the present patient (original magnification × 400. Scale bar = 50 µm). TRIM8 was expressed in the nuclei of podocytes (podocalyxin-positive cells, **B**) (arrowheads) and endothelial cells (CD34-positive cells, **C**) (arrows). Proximal and distal tubular epithelial cells also showed the nuclear expression of TRIM8 protein in the normal control specimen (**D** and **E**). TRIM8 expression was not observed in any cells in the kidney tissue obtained from the present patient (**F**–**J**). Tubules with cystic dilatation were positive for EMA but negative for CD10 (**I** and **J**).CD10, Cluster of differentiation 10; CD34, Cluster of differentiation 34; EMA, epithelial membrane antigen; TRIM8, tripartite motif containing 8

Immunohistochemical analysis were performed using the formalin-fixed paraffin-embedded kidney biopsy specimens obtained from the present patient and nine control individuals consisting of three living kidney transplantation donors who served as normal controls, five patients with primary FSGS and one patient with nephronophthisis who served as disease controls. We performed autoclave-based antigen retrieval, for 15 min at 105 °C, in Bond Epitope Retrieval Solution 2 (Leica Biosystems Newcastle, Ltd., Newcastle Upon Tyne, UK). Specimens were incubated with goat polyclonal antibody against an epitope corresponding to amino acids 540–551, at the C-terminus of human TRIM8 (Abcam, Cambridge, MA, USA; catalog no. ab4302), overnight, at a dilution of 1:500 [5]. Immunofluorescence staining, using the anti-TRIM8 antibody in normal control specimens, is shown in Fig. 3 (Fig. 3A–E). TRIM8 expression was observed in the nuclei of all glomerular cells (Fig. 3A–C). Double immunostaining with mouse anti-human podocalyxin monoclonal antibody (PHM5, Merck Millipore, Darmstadt, Germany), at a dilution of 1:100 which was used as a podocyte marker (Fig. 3B) [12] and mouse anti-human cluster of differentiation 34 (CD34) monoclonal antibody (QBEND/10, Leica Microsystems, Wetzlar, Germany), at a dilution of 1:40 which was used as an endothelial cell marker (Fig. 3C) [13] showed that TRIM8 was expressed in the nuclei of podocytes and endothelial cells, respectively. Proximal tubular cells that were identified by mouse anti-human cluster of differentiation 10 (CD10) monoclonal antibody (56C6, Leica Microsystems, Wetzlar, Germany) without dilution [14] and distal tubular cells that were identified by mouse anti-human epithelial membrane antigen (EMA) monoclonal antibody (Clone E29, Dako, Santa Clara, California, USA) without dilution [15] also showed the nuclear expression of TRIM8 protein (Fig. 3D and E). Similar findings were observed in specimens from disease controls (Fig. S1). In contrast, the present patient showed a lack of TRIM8 protein expression in any cells in the glomeruli and tubules (Fig. 3F–J). The tubules showing cystic dilatation were positive for EMA, but negative for CD10, indicating that cystic dilatation was evident in the distal tubules (Fig. 3I and J). IHC staining, using anti-SOCS1 goat polyclonal antibody (Abcam, catalog no. ab9870), at a dilution of 1:500 [5] of the kidney biopsy specimens derived from the present patient showed stronger cytoplasmic SOCS1 expression in glomerular and tubular cells than observed in control samples (Fig. S2).

## Discussion and conclusions

We reported the first patient with a de novo variant in *TRIM8* who developed nephrotic syndrome and ESRD without any neurological manifestations. Immunofluorescence examination indicated that the variant identified in the present case (p.Tyr487*) is pathogenic because TRIM8 expression was decreased in all kidney cells, consistent with a previous report [5]. Additionally, sequence analysis of mRNA of samples from the present patient confirmed the escape of the mutant allele from NMD. These findings suggested a dominant-negative effect for the heterozygous nonsense variant, which could affect the E3 ubiquitin ligase activity of TRIM8 in the kidney [5, 16].

**Fig. 4:**
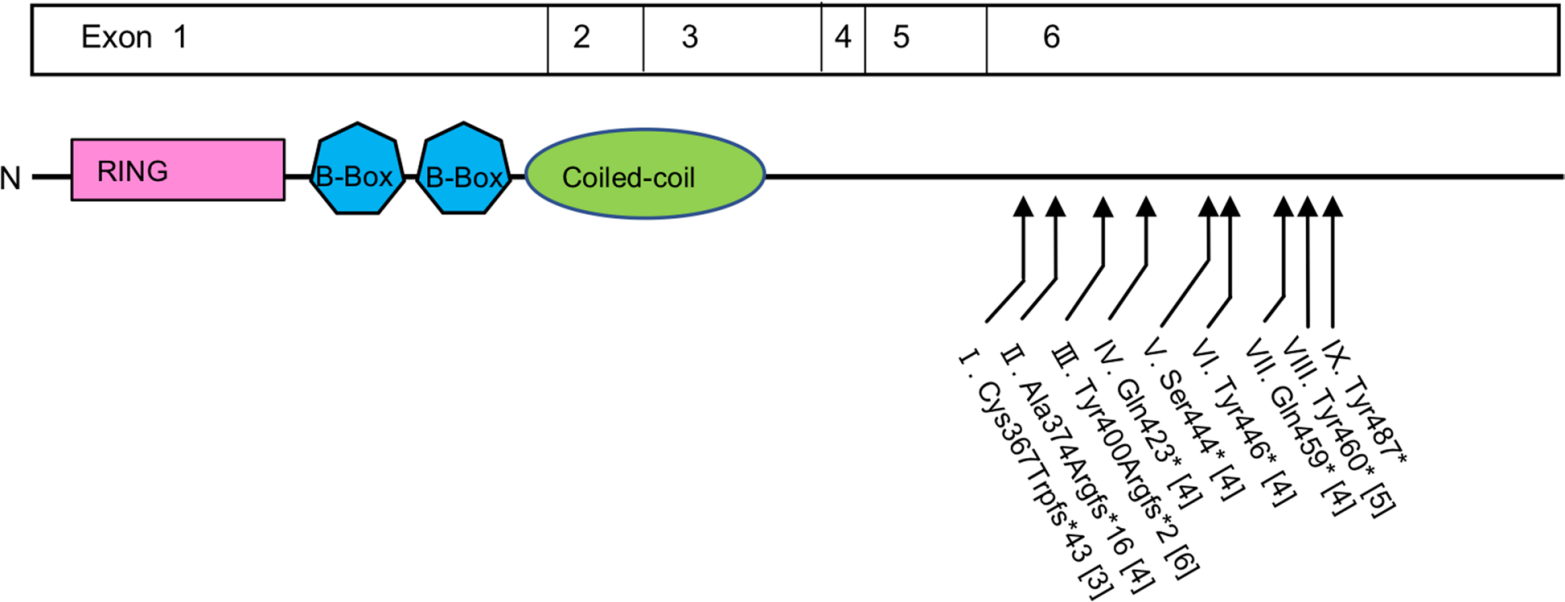
Loci of *TRIM8* variants in previously reported individuals and the present patient Proteinuria was described in patients III, V–IX [4–6]. The locus of the truncating variant in the present patient (patient IX) was closer to the C-terminal end than variants in other reported individuals [3–6]

To date, at least eight reported individuals have been associated with heterozygous truncating variants in *TRIM8*, all of whom developed epilepsy or epileptic encephalopathy, including five individuals with proteinuria (Table 1 and Fig. 4) [3–6]. The present patient did not present with any neurological manifestations such as developmental delay and epilepsy. Brain MRI showed no abnormalities in the present patient, while cortical and subcortical atrophy and cysts in white matter on MRI have been described in the literature [4]. Because whole-exome sequencing showed that alternative allele counts were approximately half of the total reads, it is unlikely that the individual was a mosaic. The locus of the truncating variant in the present patient (p.Tyr487*) was closest to the C-terminal end than variants in other reported individuals (Table 1 and Fig. 4, patient IX). Nevertheless, the variant of patient III, who had mild developmental delay and well-controlled epilepsy [6], was located more 5’ in the last exon than 5 patients with epileptic encephalopathy reported by Assoum et al. and Warren et al. [4, 5]. Therefore, a difference in the size of the truncated protein may not explain the difference in the severity of the neurological phenotype [6]. However, proteinuria tended to be documented in those individuals featuring *TRIM8* variants that affected regions closer to the C-terminal end of the TRIM8 protein (Table 1 and Fig. 4, patient III, V-IX). Three of five individuals with proteinuria developed ESRD during childhood [5, 6]. Very recently, Weng et al. reported 12 individuals with *TRIM8* variants clustering within the last exon between residues 390 and 487 of the 551 amino acid protein. All of them presented with nephrotic syndrome and neurologic disease, ten of whom showed FSGS on kidney biopsies [17]. ESRD occurred in 10 individuals at the age of 1 to 19 [17]. One of them, who had the same heterozygous variant as our patient (p.Tyr487*), presented with childhood-onset FSGS and neurological manifestations such as mild developmental delay, Tourette’s syndrome-like symptoms, and autism spectrum [17]. Collectively, these findings expanded the clinical phenotypic spectrum of *TRIM8* variants.

**Table 1:** Clinical features of the previously reported individuals with TRIM8 variants and the present patient

Patient No	Neurological features			Renal manifestations				Ref
	Age at first seizure	Seizure outcome	Other neurologic features	Proteinuria	Age at onset of proteinuria	Pathological diagnosis	Renal outcome	
I	2 months	Not controlled on medication	Intellectual disability	No	NA	NA	NA	[3]
II	5 months	Rare seizures, partially, well controlled with levetiracetam	Intellectual disability, autism spectrum disorder	No	NA	NA	NA	[4]
III	5 years	Well controlled with sodium valproate	Mild intellectual disability	Yes (Nephrotic syndrome)	2 years 2 months	FSGS	Progressed to end-stage renal disease	[6]
IV	21 months	NA	Developmental delay	No	NA	NA	NA	[4]
V	3 years and 5 months	Well controlled with levetiracetam	Developmental delay, mild head tremor, mild ataxic gait	Yes (Nephrotic syndrome)	NM	NM	Normal serum creatinine level	[4]
VI	2 years	Daily seizures	Intellectual disability, stereotypic behavior and ataxia	Yes (Nephrotic syndrome)	4 years	NM	NM	[4]
VII	21 months	Not well controlled with levetiracetam	Hypotonia, hyporeflexia, and global developmental delay	Yes	7–10 months	NM	Proteinuria resolved spontaneously	[4]
VIII	2 years and 5 months	Developed multi-focal drug resistant epilepsy	Intellectual disability and language delay	Yes (Nephrotic syndrome)	2 years 5 months	FSGS	Progressed to end-stage renal disease	[5]
IX	None	None	None	Yes (Nephrotic syndrome)	3 years	FSGS, cystic dilatation of distal tubules	Progressed to end-stage renal disease	Present case

Patient numbers are consistent with those described in Fig. 2

*NA* not applicable, *NM* not mentioned

Renal pathological assessment of the present patient showed FSGS (Fig. 1A) and cystic dilatation of the distal tubules (Fig. 1B, 3I and J), which may be associated with other manifestations observed in our patient, such as polyuria and hyposthenuria. Although no tubulointerstitial changes were described in the individual reported by Warren et al. [5], a lack of TRIM8 expression in the tubular cells was noticed in the report by Warren et al. [5] To examine the possible superimposition of nephronophthisis, we performed genetic testing and examined 83 nephronophthisis causative genes (Table S2). However, no pathogenic variants in the genes currently known to cause nephronophthisis were identified in the present patient. Further studies remain necessary to characterize tubulointerstitial changes in patients with *TRIM8* variants.

The mechanism underlying proteinuria and the cystic dilatation of tubules observed in our patient remains largely unknown. In this study, TRIM8 expression was observed in the nuclei of podocytes and tubular cells of control specimens. TRIM8 has been shown to enhance the translocation of signal transducer and activator of transcription 3 (STAT3) into the nucleus, and TRIM8 modulates STAT3 transcriptional potential in the nucleus [18]. Our study showed increased SOCS1 protein expression levels in kidney specimens from our patient, which was consistent with the report by Warren et al. Strong SOCS1 expression can inhibit cytokine signaling by binding to downstream signaling molecules such as Janus tyrosine kinases (JAK) [19], and inhibition of JAK/STAT3 activity attenuates the progression of glomerular and tubulointerstitial injury [20]. However, the associations between the TRIM8, SOCS1 protein expression, and renal manifestations remain unknown. It has been also described that TRIM8 induces p53-dependent cell cycle arrest [21]. Podocytes are terminally differentiated cells and have a limited capacity to divide. Wang et al. reported that the cellular lesion of FSGS is characterized by podocyte proliferation superimposed on sclerotic or collapsed glomerular tufts [22]. Therefore, TRIM8 dysfunction in podocytes may be associated with aberrant regulation of cell cycle, which may lead to the progression of FSGS. Cell cycle dysregulation has been also demonstrated in renal tubular epithelial cells in autosomal dominant polycystic kidney disease (ADPKD) [23]. Knockdown of *Pkd1*, a responsible gene for ADPKD, has been shown to reduce the amount of p53 and lower the fraction of cells in G1/S, suggesting that unchecked cell cycle progression is involved in the pathogenesis of tubular cyst growth [24]. Collectively, aberrant regulation of cell cycle induced by TRIM8 abnormalities may cause FSGS and the cystic dilatation of renal tubules.

In conclusion, we reported a patient with FSGS who had a de novo heterozygous *TRIM8* variant without any neurological manifestations. Our results expanded the clinical phenotypic spectrum of *TRIM8* variants CD10, cluster of differentiation 10; CD34, cluster of differentiation 10; EMA, epithelial membrane antigen; ESRD, end-stage renal disease; JAK, Janus tyrosine kinases; NMD, nonsense-mediated mRNA decay; SOCS1, Suppressor of cytokine signaling 1; STAT3, signal transducer and activator of transcription 3; TRIM8, tripartite motif containing 8; UPCR, urine protein to creatinine ratio.

## Supplementary Information

**Additional file 1: Figure S1.** Immunofluorescence staining of the disease controls. Immunofluorescence staining, using an anti-TRIM8 antibody (red) and 4′,6-diamidino-2-phenylindole (DAPI) (blue), in kidney specimens from a case with primary FSGS (a–d), and a case with nephronophthisis (e–h). TRIM8 protein was expressed in the nuclei of glomerular cells and tubular epithelial cells. (Original magnification, ×400. Scale bar = 50 µm). **Figure S2.** Immunohistochemical staining of SOCS1. Immunohistochemical staining, using an anti-SOCS1 antibody, in the present case and a normal control case. The present case showed stronger SOCS1 protein expression in the cytoplasm of glomerular and tubular epithelial cells (A) than the control case (B). (Original magnification, ×200. Scale bar = 50 µm). SOCS1, Suppressor of cytokine signaling 1. **Table S1.** The list of 65 genes which represent monogenic causes of human focal segmental glomerulosclerosis and/or steroid-resistant nephrotic syndrome. **Table S2.** The list of 83 genes which represent monogenic causes of human nephronophthisis

## Data Availability

The datasets are not publicly available but are available from the first author on reasonable request.

## Abbreviations

CD10: Cluster of differentiation 10
CD34: Cluster of differentiation 10
EMA: Epithelial membrane antigen
ESRD: End-stage renal disease
JAK: Janus tyrosine kinases
NMD: Nonsense-mediated mRNA decay
SOCS1: Suppressor of cytokine signaling 1
STAT3: Signal transducer and activator of transcription 3
TRIM8: Tripartite motif containing 8
UPCR: Urine protein to creatinine ratio
