# Supplementary material for: A novel de novo truncating TRIM8 variant associated with childhood-onset focal segmental glomerulosclerosis without epileptic encephalopathy: a case report
Source: BMC Nephrol. 2021 Dec 20;22:417. doi: 10.1186/s12882-021-02626-1 (PMC8686241; doi:10.1186/s12882-021-02626-1)
Supplement: Supplementary file 1 — Additional file 1: Figure S1. Immunofluorescence staining of the disease controls. Immunofluorescence staining, using an anti-TRIM8 antibody (red) and 4′,6-diamidino-2-phenylindole (DAPI) (blue), in kidney specimens from a case with primary FSGS (a–d), and a case with nephronophthisis (e–h). TRIM8 protein was expressed in the nuclei of glomerular cells and tubular epithelial cells. (Original magnification, ×400. Scale bar = 50 µm). Figure S2. Immunohistochemical staining of SOCS1. Immunohistochemical staining, using an anti-SOCS1 antibody, in the present case and a normal control case. The present case showed stronger SOCS1 protein expression in the cytoplasm of glomerular and tubular epithelial cells (A) than the control case (B). (Original magnification, ×200. Scale bar = 50 µm). SOCS1, Suppressor of cytokine signaling 1. Table S1. The list of 65 genes which represent monogenic causes of human focal segmental glomerulosclerosis and/or steroid-resistant nephrotic syndrome. Table S2. The list of 83 genes which represent monogenic causes of human nephronophthisis [file 12882_2021_2626_MOESM1_ESM.docx]

**Figure S1.** Immunofluorescence staining of the disease controls.

A


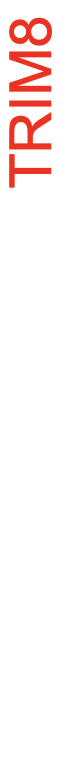

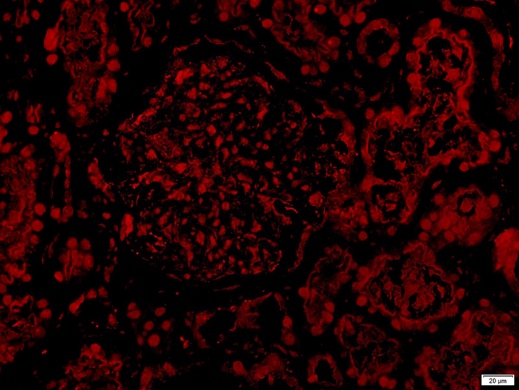

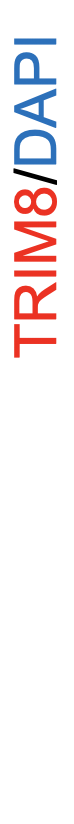

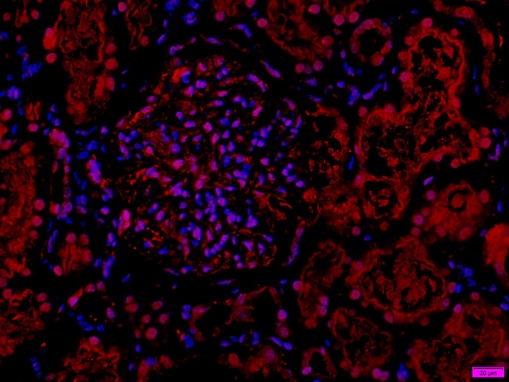

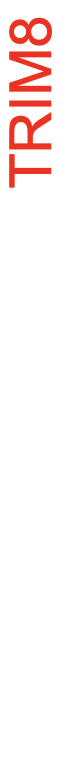
**
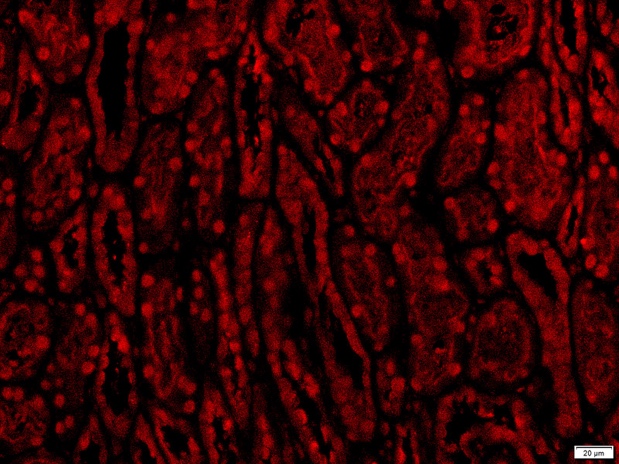
**
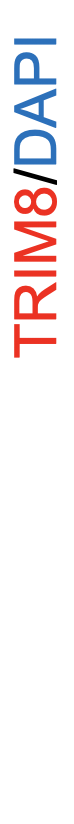

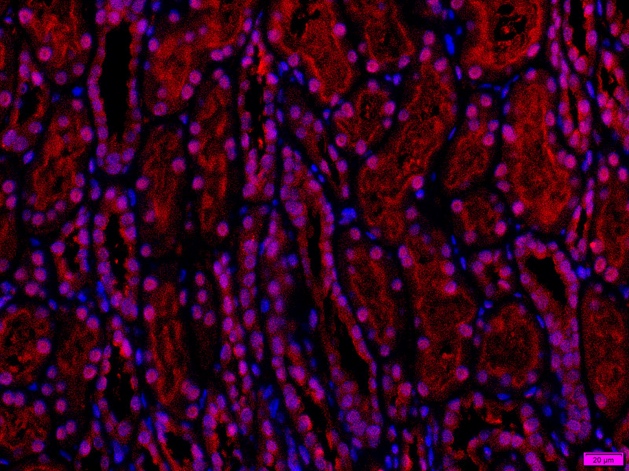


C

B

D


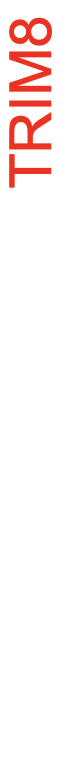

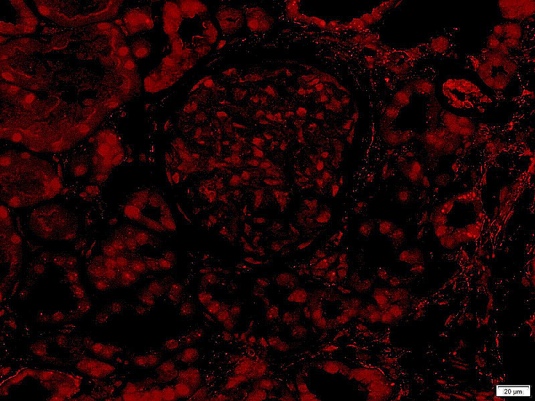

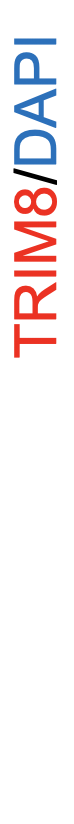

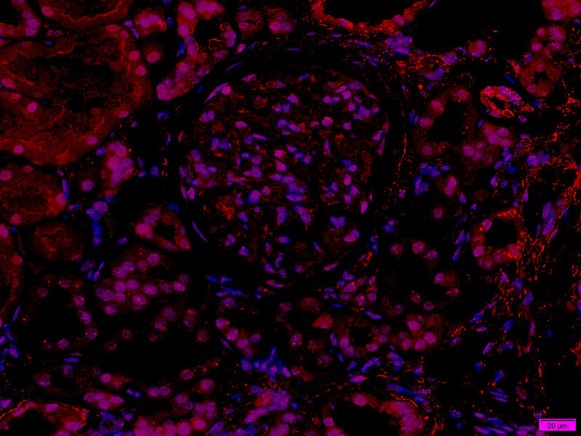

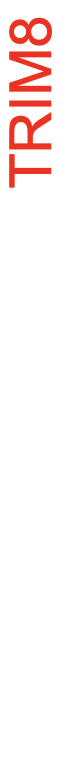

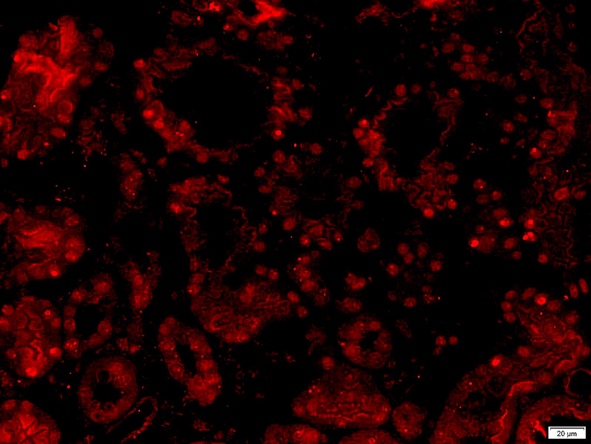

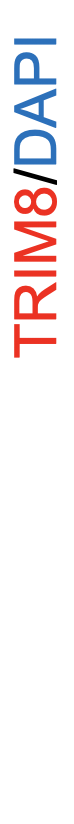

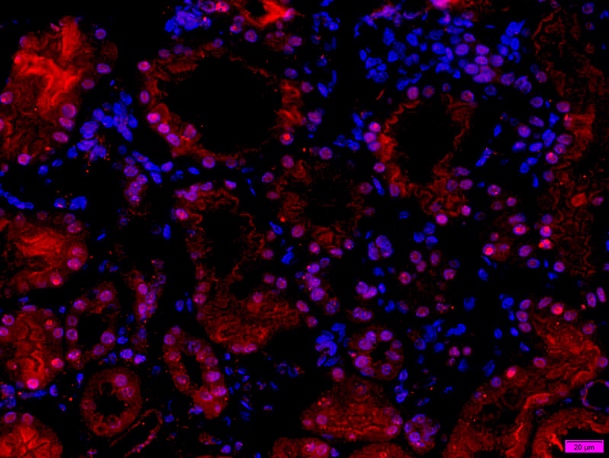


H

E

F

G

Immunofluorescence staining, using an anti-TRIM8 antibody (red) and 4′,6-diamidino-2-phenylindole (DAPI) (blue), in kidney specimens from a case with primary FSGS (a–d), and a case with nephronophthisis (e–h). TRIM8 protein was expressed in the nuclei of glomerular cells and tubular epithelial cells. (Original magnification, ×400. Scale bar = 50 µm)

**Figure S2.** Immunohistochemical staining of SOCS1.


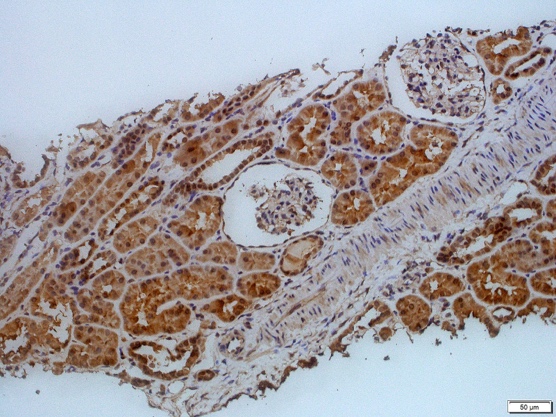

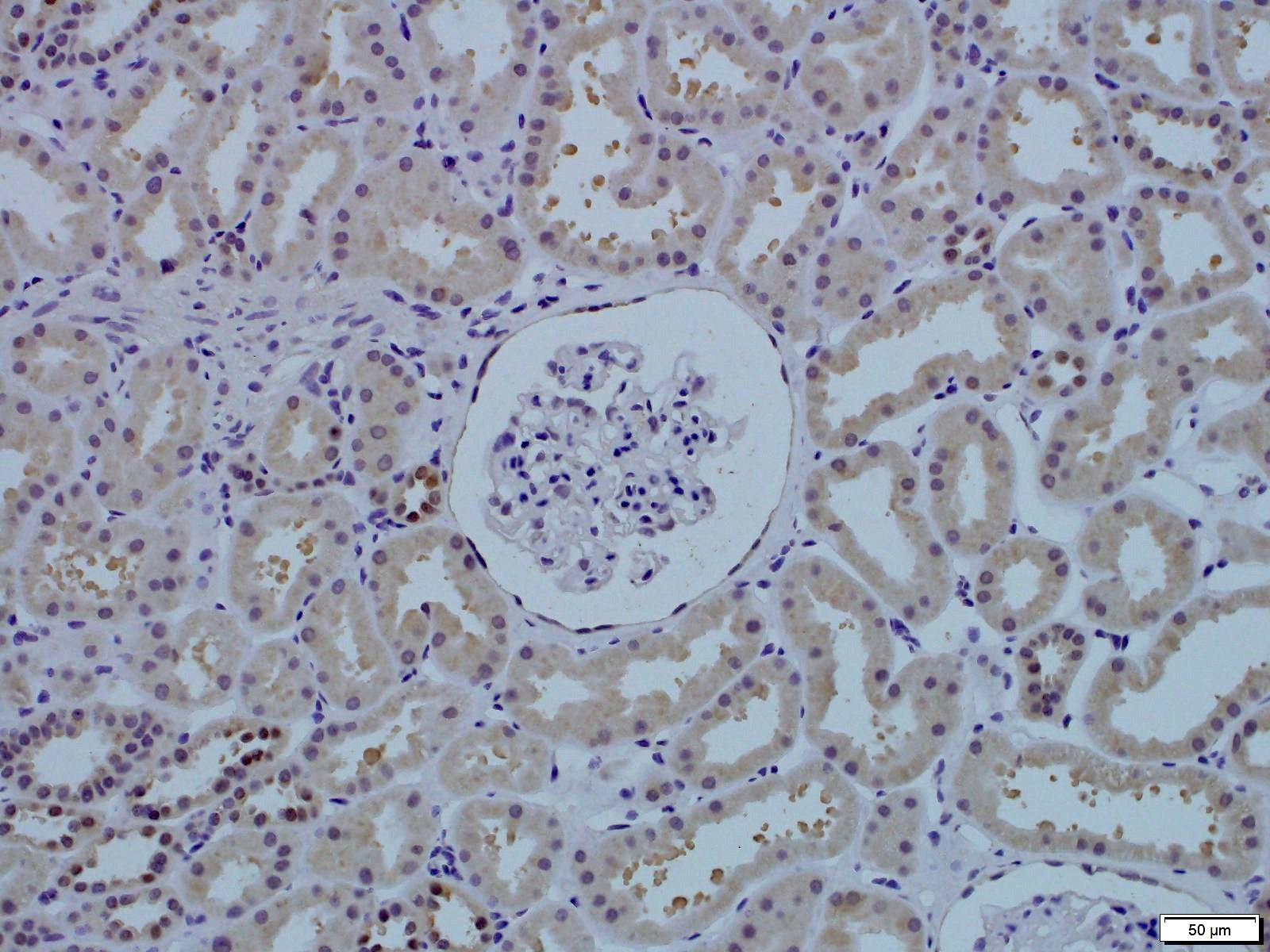


B

A

Immunohistochemical staining, using an anti-SOCS1 antibody, in the present case and a normal control case. The present case showed stronger SOCS1 protein expression in the cytoplasm of glomerular and tubular epithelial cells (**A**) than the control case (**B**). (Original magnification, ×200. Scale bar = 50 µm).

SOCS1, Suppressor of cytokine signaling 1

**Table S1.** The list of 65 genes which represent monogenic causes of human focal segmental glomerulosclerosis and/or steroid-resistant nephrotic syndrome.

| **Gene** | **Protein** | **Mode of inheritance** | **Ref.** |
| --- | --- | --- | --- |
| *ACTN4* | Alpha-actinin 4 | AD | [1] |
| *ADCK4* | AarF domain-containing kinase 4 | AR | [2] |
| *ANKFY1* | Ankyrin repeats- and FYVE domain-containing protein 1 | AR | [3] |
| *ANLN* | Actin-binding protein anillin | AD | [4] |
| *ARHGAP24* | Rho GTPase-activating protein 24 | AD | [5] |
| *ARHGDIA* | Rho GDP-dissociation inhibitor alpha | AR | [6] |
| *AVIL* | Advillin | AR | [7] |
| *CD151* | Cd151 antigen | AR | [8] |
| *CD2AP* | Cd2-associated protein | AD/AR | [9] |
| *COL4A3* | Collagen, type IV, alpha-3 | AD/AR | [10] |
| *COL4A4* | Collagen, type IV, alpha-4 | AD/AR | [10] |
| *COL4A5* | Collagen, type IV, alpha-5 | XLD | [10] |
| *COQ2* | Coenzyme q2, polyprenyltransferase | AR | [11] |
| *COQ6* | Coenzyme q6, monooxygenase | AR | [12] |
| *CRB2* | Crumbs homolog 2 | AR | [13] |
| *CUBN* | Cubilin | AR | [14] |
| *DLC1* | Deleted in liver cancer 1 | AR | [15] |
| *EMP2* | Epithelial membrane protein 2 | AR | [16] |
| *FAT1* | Fat atypical cadherin 1 | AR | [17] |
| *GAPVD1* | GTPase-activating protein and vps9 domains 1 | AR | [3] |
| *GON7* | C14orf142; chromosome 14 open reading frame 142 | AR | [18] |
| *INF2* | Inverted formin 2 | AD | [19] |
| *ITGA3* | Integrin, alpha-3 | AR | [20] |
| *ITGB4* | Integrin, beta-4 | AR | [21] |
| *ITSN1* | Intersectin 1 | AR | [15] |
| *ITSN2* | Intersectin 2 | AR | [15] |
| *KANK1* | Kn motif- and ankyrin repeat domain-containing protein 1 | AR | [22] |
| *KANK2* | Kn motif- and ankyrin repeat domain-containing protein 2 | AR | [22] |
| *KANK4* | Kn motif- and ankyrin repeat domain-containing protein 4 | AR | [22] |
| *KIRREL1* | Kin of IRRE-like protein 1 | AR | [23] |
| *LAGE3* | L antigen family, member 3 | XLR | [24] |
| *LAMA5* | Laminin, alpha-5 | AR | [25] |
| *LAMB2* | Laminin, beta-2 | AR | [26] |
| *LMX1B* | Lim homeobox transcription factor 1, beta | AD | [27] |
| *MAGI2* | Membrane-associated guanylate kinase, WW, and PDZ domain-containing 2 | AR | [15] |
| *MYO1E* | Myosin 1E | AR | [28] |
| *NPHS1* | Nephrin | AR | [29] |
| *NPHS2* | Podocin | AR | [30] |
| *NUP107* | Nucleoporin, 107-kd | AR | [31] |
| *NUP205* | Nucleoporin, 205-kd | AR | [32] |
| *NUP93* | Nucleoporin, 93-kd | AR | [32] |
| *NUP133* | Nucleoporin, 133-kd | AR | [33] |
| *NUP85* | Nucleoporin, 85-kd | AR | [33] |
| *NXF5* | Nuclear RNA export factor 5 | XLR | [34] |
| *OSGEP* | O-sialoglycoprotein endopeptidase | AR | [24] |
| *PAX2* | Paired box protein pax-2 | AD | [35] |
| *PDSS2* | Prenyl diphosphate synthase, subunit 2 | AR | [36] |
| *PLCE1* | Phospholipase c, epsilon-1 | AR | [37] |
| *PODXL* | Podocalyxin like protein | AD | [38] |
| *PTPRO* | Protein-tyrosine phosphatase, receptor-type, o | AR | [39] |
| *SCARB2* | Scavenger receptor class b, member 2 | AR | [40] |
| *SGPL1* | Sphingosine-1-phosphate lyase 1 | AR | [41] |
| *SMARCAL1* | Swi/snf-related, matrix-associated, actin-dependent regulator of chromatin, subfamily a-like protein 1 | AR | [42] |
| *TNS2(TENC1)* | Tensin 2 | AR | [15] |
| *TP53RK* | Tp53-regulating kinase | AR | [24] |
| *TPRKB* | Tp53rk-binding protein | AR | [24] |
| *TRPC6* | Transient receptor potential cation channel, subfamily c, member 6 | AD | [43] |
| *TRIM8* | Tripartite motif containing 8 | AD | [44] |
| *TTC21B* | Tetratricopeptide repeat domain-containing protein 21b | AR | [45] |
| *WDR4* | Wd repeat-containing protein 4 | AR | [46] |
| *WDR73* | Wd repeat-containing protein 73 | AR | [47] |
| *WT1* | Wilms’ tumour protein 1 | AD | [48] |
| *XPO5* | Exportin 5 | AR | [32] |
| *YRDC* | YrdC domain-containing protein | AR | [18] |
| *ZMPSTE24* | Zinc metalloproteinase ste24 | AR | [49] |

AD, autosomal dominant; AR, autosomal recessive; FSGS, focal segmental glomerulosclerosis; SRNS, steroid-resistant nephrotic syndrome; XLD, X-linked dominant; XLR, X-linked recessive

**Table S2.** The list of 83 genes which represent monogenic causes of human nephronophthisis.

| **Gene** | **Protein** | **Ref.** |
| --- | --- | --- |
| *NPHP1* | Nephrocystin-1 | [50] |
| *NPHP2/INVS* | Inversin | [51] |
| *NPHP3* | Nephrocystin-3 | [52] |
| *NPHP4* | Nephrocystin-4 | [53] |
| *NPHP5/IQCB1* | Nephrocystin-5 | [54] |
| *NPHP6/CEP290* | Centrosomal protein 290 | [55] |
| *NPHP7/GLIS2* | GLIS family zinc finger 2 | [56] |
| *NPHP8/RPGRIP1L* | Retinitis pigmentosa GTPase regulator interacting protein 1-like | [57] |
| *NPHP9/NEK8* | Never in mitosis A-related kinase 8 | [58] |
| *NPHP10/SDCCAG8* | Serologically defined colon cancer antigen 8 | [59] |
| *NPHP11/TMEM67* | Meckelin | [60] |
| *NPHP12/TTC21B* | Tetratricopeptide repeat domain 21B | [61] |
| *NPHP13/WDR19* | WD repeat domain 19 | [62] |
| *NPHP14/ZNF423* | Zinc finger protein 423 | [63] |
| *NPHP15/CEP164* | Centrosomal protein 164 | [63] |
| *NPHP16/ANKS6* | Ankyrin repeat and sterile alpha motif Domain Containing 6 | [64] |
| *NPHP17/IFT172* | Intraflagellar transport 172 | [65] |
| *NPHP18/CEP83* | Centrosomal protein 83kDa | [66] |
| *NPHP1L/XPNPEP3* | Xaa-pro aminopeptidase 3 | [67] |
| *NPHP2L/SLC41A1* | Solute carrier family 41 member 1 | [68] |
| *AHI1* | Abelson-helper integration site-1 | [69] |
| *ALMS1* | Alstrom syndrome protein 1 | [70] |
| *ARL13B* | ADP-ribosylation factor-like protein 13B | [71] |
| *ARL6* | ADP-ribosylation factor like GTPase 6 | [72] |
| *ATXN10* | Ataxin 10 | [73] |
| *B9D1* | B9 domain containing 1 | [74] |
| *B9D2* | B9 domain containing 2 | [74] |
| *BBIP1* | BBSome interacting protein 1 | [75] |
| *BBS1* | Bardet–Biedl syndrome-1 | [76] |
| *BBS10* | Bardet–Biedl syndrome-10 | [76] |
| *BBS12* | Bardet–Biedl syndrome-12 | [77] |
| *BBS2* | Bardet–Biedl syndrome-2 | [78] |
| *BBS4* | Bardet–Biedl syndrome-4 | [79] |
| *BBS5* | Bardet–Biedl syndrome-5 | [80] |
| *BBS7* | Bardet–Biedl syndrome-7 | [81] |
| *BBS9* | Bardet–Biedl syndrome-9 | [82] |
| *CEP83* | Centrosomal protein 83 | [83] |
| *CEP104* | Centrosomal protein 104 | [84] |
| *CEP164* | Centrosomal protein 164 | [85] |
| *CEP 41* | Centrosomal protein of 41 kDa | [86] |
| *C14ORF179* | intraflagellar transport 43 | [87] |
| *C5orf42* | Ciliogenesis and planar polarity effector 1 | [88] |
| *C8orf37* | Chromosome 8 open reading frame 37 | [89] |
| *CC2D2A* | Coiled-Coil and C2 domain containing 2A | [88] |
| *CSPP1* | Centrosome and spindle pole associated protein 1 | [90] |
| *DCDC2* | Doublecortin domain containing 2 | [91] |
| *DYNC2H1* | Dynein cytoplasmic 2 heavy chain 1 | [92] |
| *EVC* | Ellis-van creveld syndrome | [93] |
| *EVC2* | Ellis-van creveld syndrome 2 | [93] |
| *FAN1* | Fanconi-associated nuclease 1 | [94] |
| *IFT27* | Intraflagellar transport 27 | [95] |
| *IFT74* | Intraflagellar transport 74 | [95] |
| *IFT80* | Intraflagellar transport 80 | [96] |
| *IFT122* | Intraflagellar transport 122 | [97] |
| *IFT140* | Intraflagellar transport 140 | [98] |
| *IFT43* | Intraflagellar transport 43 | [97] |
| *IFT52* | Intraflagellar transport 52 | [97] |
| *INPP5E* | Inositol polyphosphate-5-phosphatase E | [99] |
| *KIAA0556* | KIAA0556 | [100] |
| *KIAA0586* | KIAA0586 | [101] |
| *KIF14* | Kinesin family member 14 | [102] |
| *KIF7* | Kinesin family member 7 | [103] |
| *LZTFL1* | Leucine zipper transcription factor like 1 | [104] |
| *MAPKBP1* | Mitogen-activated proteinkinase binding protein 1 | [105] |
| *MKKS* | McKusick-Kaufman syndrome | [106] |
| *OFD1* | Oral-facial-digital type I | [107] |
| *PDE6D* | Phosphodiesterase 6D | [108] |
| *RPGRIP1L* | RPGR-interacting protein 1-like | [109] |
| *SLC41A1* | Solute carrier family 41, member 1 | [110] |
| *TCTN1* | Tectonic family member 1 | [111] |
| *TCTN2* | Tectonic family member 2 | [112] |
| *TMEM138* | Transmembrane protein 138 | [113] |
| *TMEM216* | Transmembrane protein 216 | [113] |
| *TMEM231* | Transmembrane protein 231 | [114] |
| *TMEM237* | Transmembrane protein 237 | [114] |
| *TMEM67* | Transmembrane protein 67 | [115] |
| *TRAF3IP1* | TRAF3 interacting protein 1 | [116] |
| *TRIM32* | Tripartite motif-containing protein 32 | [117] |
| *WDR19* | WD repeat domain 19 | [97] |
| *WDR34* | WD repeat domain 34 | [118] |
| *WDR35* | WD repeat domain 35 | [97] |
| *XPNPEP3* | Xaa-pro aminopeptidase 3 | [119] |
| *ZNF423* | Zinc finger protein 423 | [85] |

**References**

[1] Kaplan JM, Kim SH, North KN, et al. Mutations in ACTN4, encoding alpha-actinin-4, cause familial focal segmental glomerulosclerosis. Nat Genet. 2000;24:251–6.

[2] Ashraf S, Gee HY, Woerner S, et al. ADCK4 mutations promote steroid-resistant nephrotic syndrome through CoQ10 biosynthesis disruption. Clin Invest. 2013;23:5179–89.

[3] Hermle T, Schneider R, Schapiro D, et al. GAPVD1 and ANKFY1 Mutations Implicate RAB5 Regulation in Nephrotic Syndrome. J Am Soc Nephrol. 2018;29:2123–38.

[4] Gbadegesin RA, Hall G, Adeyemo A, et al. Mutations in the gene that encodes th F-actin binding protein anillin cause FSGS. J Am Soc Nephrol. 2014;25:1991–2002.

[5] Akilesh S, Suleiman H, Yu H, et al. Arhgap24 inactivates Rac1 in mouse podocytes, and a mutant form is associated with familial focal segmental glomerulosclerosis. J Clin Invest. 2000;121:4127–37.

[6] Gee HY. Saisawat P, Ashraf S, et al. ARHGDIA mutations cause nephrotic syndrome via defective RHO GTPase signaling. J Clin Invest. 2013;123:3243–53.

[7] Rao J, Ashraf S, Tan W, et al. Adillin acts upstream of phospholipase C ε1 in steroid-resistant nephrotic syndrome. J Clin Invest. 2017;127:4257–69.

[8] Crew VK, Burton N, Kagan A, et al. CD151, the first member of the tetrapanin (TM4) superfamily detected on erythrocytes, is essential for the correct assembly of human basement membranes in kidney and skin. Blood. 2004;104 2217–23.

[9] Kim JM, Wu H, Green G, et al. CD2-associated protein haploinsufficiency is linked to glomerular disease susceptibility. Science. 2003;300:1298–300.

[10] Gast C, Pengelly RJ, Lyon M, et al. Collagen (COL4A) mutations are the most frequent mutations underlying adult focal segmental glomerulosclerosis. Nephrol Dial Transplant. 2016;31:961–70.

[11] Diomedi-Camassei F, Giandomenico SD, Santorelli FM, et al. COQ2 nephropathy: a newly dscribed inherited mitochondriopashy with primary renal involvement. J Am Soc Nephrol. 2007;18:2773–80.

[12] Heeringa SF, Chernin G, Chaki Mounita, et al. COQ6 mutations in human patients produce nephrotic syndrome with sensorineural deafness. J Clin Invest. 2011;121:2013–24.

[13] Ebarasi L, Ashraf S, Bierzynska A, et al. Defects of CRB2 cause steroid-resistant nephrotic syndrome. Am J Hum Genet. 2015;96:153–61.

[14] Bedin M, Boyer O, Servais A, et al. Human C-terminal CUBN variants associate with chronic proteinuria and normal renal function. J Clin Invest. 2020;130:335–44.

[15] Ashraf S, Kudo H, Rao J, et al. Mutations in six nephrosis genes delineate a pathogenic pathway amenable to treatment. Nat Commun. 2018;9:1960.

[16] Gee HY, Ashraf S, Wan X, et al. Mutations in EMP2 cause childhood-onset nephrotic syndrome. Am J Hum Genet. 2014;94:884–90.

[17] Gee HY, Sadowski CE, Aggarwal PK, et al. FAT1 mutations cause a glomerulotubular nephropathy. Nat Commun. 2016;7:10822.

[18] Arrondel C, Missoury S, Snoek R, et al. Defects in t6 A tRNA modification due to GON7 and YRDC mutations lead to Galloway-Mowat syndrome. Nat Commun. 2019;10:3967.

[19] Brown EJ, Schlöndorff JS, Becker DJ, et al. Mutations in the formin gene INF2 cause focal segmental glomerulosclerosis. Nat Genet. 2010;42:72–6.

[20] Yalcin EG, He Y, Orhan D, et al. Crucial role of posttranslational modifications of integrin α3 in interstitial lung disease and nephrotic sydrome. Hum Mol Genet. 2015;24:3679–88.

[21] Kambham N, Tanji N, Seigle RL, et al. Congenital focal segmental glomerulosclerosis associated with beta4 integrin mutation and epidermolysis bullosa. Am J Kidney Dis. 2000;36:190–6.

[22] Gee HY, Zhang F, Ashraf S, et al. KANK deficiency leads to podocyte dysfunction and nephrotic syndrome. J Clin Invest. 2015;125:2375–84.

[23] Solanki AK, Widmeier Eugen, Arif E, et al/ Mutations in KIRREL1, a slit diaphragm component, cause steroid-resistant nephrotic syndrome. Kidney Int. 2019:96:883–9.

[24] Braun DA, Rao J, Mollet G, et al. Mutations in KEOPS-complex genes cause nephrotic syndrome with primary microcephaly. Nat Genet. 2017;49:1529–38.

[25] Braun DA, Warejko JK, Ashraf S, et al. Genetic variants in the LAMA5 gene in pediatric nephrotic syndrome. Nephrol Dial Transplant. 2019;34:485–93.

[26] Zenker M, Aigner T, Wendler O, et al. Human laminin beta2 deficiency causes congenital nephrosis with mesangial sclerosis and distinct eye abnormalities. Hum Mol Genet. 2004;13:2625–32.

[27] Boyer O, woerner S, Yang F, et al. LMX1B mutations cause hereditary FSGS without extrarenal involvement. J Am Soc Nephrol. 2013;24:1216–22.

[28] Mele C, Latropoulos P, Donadelli R, et al. MYO1E mutations and childhood familial focal segmental glomerulosclerosis. N Engl J Med. 2011;365:295–306.

[29] Santίn S, Garcίa-Maset R, Ruίz P, et al. Nephrin mutations cause childhood- and adult- onset focal segmental glomerulosclerosis. Kidney Int. 2009;76:1268–76.

[30] Boute N, Gribouval O, Rosellli S, et al. NPHS2, encoding the glomerular protein podocin, is mutated in autosomal recessive steroid-resistant nephrotic syndrome. Nat Genet. 2000;24:349–54.

[31] Miyake N, Tsukaguchi H, Koshimizu E, et al. Biallelic mutations in nuclear pore complex subunit NUP107 cause early-childhood-onset steroid-resistant nephrotic syndrome. Am J Hum Genet. 2015;97:555–66.

[32] Braun DA, Sadowski CE, Kohl S, et al: Mutations in nuclear pore genes NUP93, NUP205 and XPO5 cause steroid-resistant nephrotic syndrome. Nat Genet. 2016;48:457–65.

[33] Braun DA, Lovric S, Schapiro D, et al. Mutations in multiple components of the nuclear pore complex cause nephrotic syndrome. J Clin Invest. 2018;128:4313–28.

[34] Esposito T, Lea RA, Maher BH, et al. Unique X-linked familial FSGS with co-segregating heart block disorder is associated with a mutation in the NXF5 gene. Hum Mol Genet. 2018;22:3654–66.

[35] Barua M, Stellacci E, Stella L, et al. Mutations in PAX2 associated with adult-onset FSGS. J Am Soc Nephrol. 2014;25:1942–53.

[36] Lványi B, Rácz GZ, Gál P, et al. Diffuse mesangial sclerosis in a PDSS2 mutation-induced coenzyme Q10 deficiency. Pediatr Nephrol. 2018;33:439–46.

[37] Hinkes B, Wiggins RC, Gbadegesin R, et al. Positional cloning uncovers mutations in PLCE1 responsible for a nephrotic syndrome variant that may be reversible. Nat Genet. 2006;38:1397–405.

[38] Lin FJ, Yao L, Hu XQ, et al. First identification of PODXL nonsense mutations in autosomal dominant focal segmental glomerulosclerosis. Clin Sci. 2019;133:9–21.

[39] Ozaltin F, Lbsirlioglu T, Taskiran EZ, et al. Disruption of PTPRO causes childhood-onset nephrotic syndrome. Am J Hum Genet. 2011;89:139–47.

[40] Berkovic SF, Dibbens LM, Oshlack A, et al. Array-based gene discovery with three unrelated subjects shows SCARB2/LIMP-2 deficiency causes myoclonus epilepsy and glomerulosclerosis. Am J Hum Genet. 2008;82:673–84.

[41] Lovric S, Goncalves S, Gee HY, et al. Mutations in sphingosine-1-phosphate lysase cause nephrotis with ichthyosis and adrenal insufficiency. J Clin Invest. 2017;127:912–28.

[42] Lipska-Ziętkiewicz BS, Gellermann J, Boyer O, et al. Low renal but high extrarenal phenotype variability in Schimke immuno-osseous dysplasia. PLoS One. 2017;12:e0180926.

[43] Winn MP, Conlon PJ, Lynn KL, et al. A mutation in the TRPC6 cation channel causes familial focal segmental glomerulosclerosis. Science. 2005;308:1801–4.

[44] Warren M, Takeda M, Partikian A, et al. Association of a De Novo Nonsense Mutation of the TRIM8 Gene With Childhood-Onset Focal Segmental Glomerulosclerosis. Pediatr Nephrol. 2020;35:1129–32.

[45] Cong EH, Bizet AA, Boyer O, et al. A homozygous misssense mutation in the ciliary gene TTC21B causes familial FSGS. J Am Soc Nephrol. 2014;25:2435–43.

[46] Braun DA, Shril S, Sinha A, et al. Mutations in WDR4 as a new cause of Galloway-Mowat syndrome. Am J Med Genet A. 2018;176:2460–5.

[47] Colin E, Con EH, Mollet G, et al. Loss-of-function mutations in WDR73 are responsible for microcephaly and steroid-resistant nephrotic syndrome: Galloway-Mowat syndrome. Am J Hum Genet. 2014;95:637–48.

[48] Lipska BS, Ranchin B, Latropoulos P, et al. Genotype-phenotype associations in WT1 glomerulopathy. Kidney Int. 2014;85:1169–78.

[49] Agarwal AK, Zhou XJ, Hall RK, et al. Focal segmental glomerulosclerosis in patients with mandibuloacral dysplasia owing to ZMPSTE24 deficiency. J Investig Med. 2006;54:208–13.

[50] Hildebrandt F, Otto E, Rensing C, et al. A novel gene encoding an SH3 domain protein is mutated in nephronophthisis type 1. Nat Genet. 1997;17:149–53.

[51] Otto EA, Schermer B, Obara T, et al. Mutations in INVS encoding inversin cause nephronophthisis type 2, linking renal cystic disease to the function of primary cilia and left-right axis determination. Nat Genet. 2003;34:413–20.

[52] Olbrich H, Fliegauf M, Hoefele J, et al. Mutations in a novel gene, NPHP3, cause adolescent nephronophthisis, tapeto-retinal degeneration and hepatic fibrosis. Nat Genet. 2003;34:455–9.

[53] Otto E, Hoefele J, Ruf R, et al.A gene mutated in nephronophthisis and retinitis pigmentosa encodes a novel protein, nephroretinin, conserved in evolution. Am J Hum Genet. 2002;71:1161–7.

[54] Otto EA, Loeys B, Khanna H, et al. Nephrocystin-5, a ciliary IQ domain protein, is mutated in Senior-Loken syndrome and interacts with RPGR and calmodulin. Nat Genet. 2005;37:282–8.

[55] Valente EM, Silhavy JL, Brancati F, et al. Mutations in CEP290, which encodesa centrosomal protein, cause pleiotropic forms of Joubert syndrome. Nat Genet. 2006;38:623–5.

[56] Pinto K, Chetty R. Gene of the month: GLIS1-3. J Clin Pathol 2020; 22jclinpath-2020-206859. doi: 10.1136/jclinpath-2020-206859.

[57] Khanna H, Davis EE, Murga-Zamalloa CA et al. A common allele in RPGRIP1L is a modifier of retinal degeneration in ciliopathies. Nat Genet. 2009;41:739– 45.

[58] Otto EA, Trapp ML, Schultheiss UT et al. NEK8 mutations affect ciliary and centrosomal localization and may cause nephronophthisis. J Am Soc Nephrol. 2008;19:587–92.

[59] Tay SA, Vincent AL. Senior-Løken syndrome and intracranial hypertension. Ophthalmic Genet. 2020; 41: 354-7. doi:10.1080/13816810.2020.1766086.

[60] Otto EA, Tory K, Attanasio M, et al. Hypomorphic Mutations in Meckelin (MKS3/TMEM67) cause Nephronophthisis with Liver Fibrosis (NPHP11). J Med Genet 2009;46:663–70.

[61] M Abo El Fotoh WM, Al-Fiky AF. A Compound Heterozygous Mutation in the Ciliary Gene TTC21B Causes Nephronophthisis Type 12. J Pediatr Genet. 2020;9:198–202.

[62] Bredrup C, Saunier S, Oud MM, et al. Ciliopathies with skeletal anomalies and renal insufficiency due to mutations in the IFT-A gene WDR19. Am J Hum Genet. 2011;89:634–43.

[63] Chaki M, Airlk R, Ghosh AK, et al. Exome capture reveals ZNF423 and CEP164 mutations, linking renal ciliopathies to DNA damage response signaling. Cell. 2012;150:533–48.

[64] Schlimpert M, Lagies S, Budnyk V, et al. Metabolic Phenotyping of Anks3 Depletion in mIMCD-3 cells - a Putative Nephronophthisis Candidate. Sci Rep. 2018;8:9022.

[65] Halbritter J, Bizet AA, Schmidts M, et al. Defects in the IFT-B component IFT172 cause Jeune and Mainzer-Saldino syndromes in humans. Am J Hum Genet. 2013;93:915–25.

[66] Failler M, Gee HY, Krug P, et al. Mutations of CEP83 cause infantile nephronophthisis and intellectual disability. Am Hum Genet. 2014;94:905–14.

[67] O'Toole JF, Liu Y, Davis EE, et al. Individuals with mutations in XPNPEP3, which encodes a mitochondrial protein, develop a nephronophthisis-like nephropathy. J Clin Invest. 2010;120:791–802.

[68] Hurd TW, Otto EA, Mishima E, et al. Mutation of the Mg2+ Transporter SLC41A1 Results in a Nephronophthisis-Like Phenotype. J Am Soc Nephrol. 2013;24:967–77.

[69] Doering JE, Kane K, Hsiao YC, et al. Species differences in the expression of AHI1, a protein implicated in the neurodevelopmental disorder Joubert syndrome, with preferential accumulation to stigmoid bodies. J comp neurol. 2008;511:238–56.

[70] Marshall JD, Hinman EG, Collin GB, et al. Spectrum of ALMS1 variants and evaluation of genotype-phenotype correlations in Alstrom syndrome. Hum Mutat. 2007;28:1114–23.

[71] Otto EA, Ramaswami G, Janssen S, et al. Mutation analysis of 18 nephronophthisis associated ciliopathy disease genes using a DNA pooling and next generation sequencing strategy. J Med Genet. 2011;48:105–16.

[72] Maria M, Lamers IJ, Schmidts M, et al. Genetic and clinical characterization of Pakistani families with Bardet-Biedl syndrome extends the genetic and phenotypic spectrum. Sci Rep. 2016;6:34764. doi: 10.1038/srep34764.

[73] Sang L, Miller JJ, Corbit KC, et al. Mapping the NPHP-JBTS-MKS protein network reveals ciliopathy disease genes and pathways. Cell. 2011;145:513–28.

[74] Williams CL, Winkelbauer ME, Schafer JC, et al. Functional redundancy of the B9 proteins and nephrocystins in Caenorhabditis elegans ciliogenesis. Mol Biol Cell. 2008;19:2154–68.

[75] Scheidecker S, Etard C, Pierce NW, et al. Exome sequencing of Bardet-Biedl syndrome patient identifies a null mutation in the BBSome subunit BBIP1 (BBS18). J Med Genet. 2014;51:132–6.

[76] Forsythe E, Beales PL. Bardet-Biedl syndrome. Eur J Hum Genet. 2013;21:8–13.

[77] Fattahi Z, Rostami P, Najmabadi A, et al. Mutation profile of BBS genes in Iranian patients with Bardet – Biedl syndrome: genetic characteriza- tion and report of nine novel mutations in five BBS genes. J Hum Genet. 2014;59:368–75.

[78] Fedick A, Jalas C, Abeliovich D, et al. Carrier frequency of two BBS2 mutations in the Ashkenazi population. Clin Genet. 2014;85:578–82.

[79] Mykytyn K, Mullins RF, Andrews M et al. Bardet-Biedl syndrome type4 (BBS4)-null mice implicate Bbs4 in agella formation but not globalcilia assembly. Proc Natl Acad Sci. 2004;101:8664–9.

[80] Li JB, Gerdes JM, Haycraft CJ et al. Comparative genomics identifies a flagellar and basal body proteome that includes the BBS5 human disease gene. Cell. 2004;117:541–52.

[81] Badano JL, Kim JC, Hoskins BE et al. Heterozygous mutations inBBS1, BBS2 and BBS6 have a potential epistatic effect on Bardet Biedl patients with two mutations at a second BBS locus. Hum Mol Genet. 2003;12:1651–9.

[82] Muller J, Stoetzel C, Vincent MC et al. Identification of 28 novel mutations in the Bardet–Biedl syndrome genes: the burden of private mutations in an extensively heterogeneous disease. Hum Genet. 2010;127:583–93.

[83] Failler M, Gee HY, Krug P, et al. Mutations of CEP83 Cause Infantile Nephronophthisis and Intellectual Disability. Am J Hum Genet. 2014;94:905–14.

[84] Luo M, Cao L, Cao Z, et al. Whole exome sequencing reveals novel CEP104 mutations in a Chinese patient with Joubert syndrome. Mol Genet Genomic Med. 2019;7:e1004.

[85] Stokman MF, Zwaag B, Kar N, et al. Clinical and genetic analyses of a Dutch cohort of 40 patients with a nephronophthisis-related ciliopathy. Pediatr Nephrol. 2018;33:1701–12.

[86] Lee JE, Silhavy JL, Zaki MS, et al. CEP41 is mutated in Joubert syndrome and is required for tubulin glutamylation at the cilium. Nat Genet. 2012;44:193–9.

[87] Duran I, Taylor SP, Zhang W, et al. Mutations in IFT-A satellite core component genes IFT43 and IFT121 produce short rib polydactyly syndrome with distinctive campomelia. Cilia. 2017;6:7.

[88] Srour M, Hamdan FF, Schwartzentruber JA, et al. Mutations in TMEM231 cause Joubert syndrome in French Canadians. J Med Genet. 2012;49:636–41.

[89] Heon E, Kim G, Qin S, Garrison JE, et al. Mutations in C8ORF37 cause Bardet Biedl syndrome (BBS21). Hum Mol Genet. 2016; 25:228–94.

[90] Akizu N, Silhavy JL, Rosti RO, et al. Mutations in CSPP1 lead to classical Joubert syndrome. Am J Hum Genet. 2014;94:80–6.

[91] Schueler M, Braun DA, Chandrasekar G, et al. DCDC2 mutations cause a renal-hepatic ciliopathy by disrupting Wnt signaling. Am J Hum Genet. 2015;96:81–92.

[92] Liang N, Jiang X, Zeng L, et al. 28 novel mutations identified from 33 Chinese patients with cilia-related kidney disorders. Clin Chim Acta. 2020;501:207–15.

[93] Moudgil A, Bagga A, Kamil ES, et al. Nephronophthisis associated with Ellis-van Creveld syndrome Pediatr Nephrol. 1998;12:20–2.

[94] Zhou W, Otto EA, Cluckey A, et al. FAN1 mutations cause karyomegalic interstitial nephritis, linking chronic kidney failure to defective DNA damage repair. Nat Genet. 2012;44:910–5.

[95] Schaefer E, Delvallée C, Mary L, et al. Identification and Characterization of Known Biallelic Mutations in the IFT27 (BBS19) Gene in a Novel Family With Bardet-Biedl Syndrome. Front Genet. 2019;10:21.

[96] Halbritter J, Bizet AA, Schmidts M, et al. Defects in the IFT-B component IFT172 cause Jeune and Mainzer-Saldino syndromes in humans. Am J Hum Genet. 2013;93:915–25.

[97] Walczak-Sztulpa J, Wawrocka A, Sobierajewicz A, et al. Intrafamilial phenotypic variability in a Polish family with Sensenbrenner syndrome and biallelic WDR35 mutations. Am J Med Genet. 2017;173:1364–8.

[98] Schmidts M, Frank V, Eisenberger T, et al. Combined NGS approaches identify mutations in the intraflagellar transport gene IFT140 in skeletal ciliopathies with early progressive kidney Disease. Hum Mutat. 2013;34:714–24.

[99] Hardee I, Soldatos A, Davids M, et al. Defective ciliogenesis in INPP5E-related Joubert syndrome. Am J Med Genet A. 2017;173:3231–7.

[100] Roosing S, Rosti RO, Rosti B, et al. Identification of a homozygous nonsense mutation in KIAA0556 in a consanguineous family displaying Joubert syndrome. Hum Genet. 2016;135:919–21.

[101] Alby C, Piquand K, Huber C, et al. Mutations in KIAA0586 Cause Lethal Ciliopathies Ranging from a Hydrolethalus Phenotype to Short-Rib Polydactyly Syndrome. Am J Hum Genet. 2015;97:311–8.

[102] Reilly ML, Stokman MF, Magry V, et al. Loss-of-function mutations in KIF14 cause severe microcephaly and kidney development defects in humans and zebrafish. Hum Mol Genet. 2019;28:778–95.

[103] Asadollahi R, Strauss JE, Zenker M, et al. Clinical and experimental evidence suggest a link between KIF7 and C5orf42-related ciliopathies through Sonic Hedgehog signaling. Eur J Hum Genet. 2018;26:197–209.

[104] Marion V, Stutzmann F, Gérard M, et al. Exome sequencing identifies mutations in LZTFL1, a BBSome and smoothened trafficking regulator, in a family with Bardet--Biedl syndrome with situs inversus and insertional polydactyly. J Med Genet. 2012;49:317–21.

[105] Schonauer R, Jin W, Ertel A, et al. Novel Nephronophthisis-associated variants reveal functional importance of MAPKBP1 dimerization for centriolar recruitment. Kidney Int. 2019;4:30632–3. doi: 10.1016/j.kint.2020.05.027.

[106] Yamamura T, Morisada N, Nozu K, et al. Rare renal ciliopathies in non-consanguineous families that were identified by targeted resequencing. Clin Nephrol. 2017;21:136–42.

[107] Ferrante MI, Giorgio G, Feather SA, et al. Identification of the gene for oral-facial-digital type I syndrome. Am J Hum Genet. 2001;68:569–76.

[108] Humbert MC, Weihbrecht K, Searby CC, et al. ARL13B, PDE6D, and CEP164 form a functional network for INPP5E ciliary targeting. Proc Natl Acad Sci USA. 2012;109:19691–6.

[109] Delous M, Baala L, Salomon R, et al. The ciliary gene RPGRIP1L is mutated in cerebello-oculo-renal syndrome (Joubert syndrome type B) and Meckel syndrome. Nat Genet. 2007;39:875–81.

[110] Amiri FS, Kariminejad A. Juvenile nephronophthisis and dysthyroidism: a rare association. CEN Case Rep. 2017;6:98–104.

[111] Garcia-Gonzalo FR, Corbit KC, Sirerol-Piquer MS, et al. A transition zone complex regulates mammalian ciliogenesis and ciliary membrane composition. Nat Genet. 2011;43:776–84.

[112] Zhang M, Chang Z, Tian Y, et al. Two novel TCTN2 mutations cause Meckel-Gruber syndrome. J Hum Genet. 2020, doi: 10.1038/s10038-020-0804-0.

[113] Bruel AL, Franco B, Duffourd Y, et al. Fifteen years of research on oral-facial-digital syndromes: From 1 to 16 causal genes. J Med Genet. 2017;54:371–80.

[114] Lambacher NJ, Bruel AL, van Dam TJ, et al. TMEM107 recruits ciliopathy proteins to subdomains of the ciliary transition zone and causes Joubert syndrome. Nat Cell Biol. 2016;18:122–31.

[115] Williams CL, Li C, Kida K, et al. MKS and NPHP modules cooperate to establish basal body/transition zone membrane associations and ciliary gate function during ciliogenesis. J Cell Biol. 2011;192:1023–41.

[116] Bizet AA, Becker-Heck A, Ryan R, et al. Mutations in TRAF3IP1/IFT54 reveal a new role for IFT proteins in microtubule stabilization. Nat Commun. 2015;6:1–14.

[117] Ramachandran H, Schäfer T, Kim Y, et al. Interaction with the Bardet-Biedl gene product TRIM32/BBS11 modifies the half-life and localization of Glis2/NPHP7. J Biol Chem. 2014;289:8390–401.

[118] Schmidts M, Vodopiutz J, Christou-Savina S, et al. Mutations in the gene encoding IFT dynein complex component WDR34 cause Jeune asphyxiating thoracic dystrophy. Am J Hum Genet. 2013;93:932–44.

[119] Alizadeh R, Jamshidi S, Keramatipour M, et al. Whole Exome Sequencing Reveals a XPNPEP3 Novel Mutation Causing Nephronophthisis in a Pediatric Patient. Iran Biomed J. 2020, doi: 10.29252/ibj.24.6.400.

*BMC Nephrology*: A novel *de novo* truncating TRIM8 mutation associated with childhood-onset focal segmental glomerulosclerosis without epileptic encephalopathy: Case report

Authors: Yoko Shirai, Kenichiro Miura, Naoto Kaneko, Kiyonobu Ishizuka, Amane Endo, Taeko Hashimoto, Shoichiro Kanda, Yutaka Harita, Motoshi Hattori.

Affiliations: Department of Pediatric Nephrology, Tokyo Women’s Medical University, Department of Pediatrics; Department of Pediatrics and Adolescent Medicine, Juntendo University Graduate School of

Medicine; Department of Pediatrics, Yamagata University School of Medicine; Department of Pediatrics, Graduate School of Medicine, The University of Tokyo.

Address Correspondence to: Motoshi Hattori (hattori@twmu.ac.jp)
